# Clinical and genetic characteristics associated with dual-positive gene variations

**DOI:** 10.3389/fnins.2025.1516589

**Published:** 2025-07-22

**Authors:** Li Wang, Jinghe Shi, Xiaojing Yin, Pingyun Qiao, Fuwei Li, Weixing Feng

**Affiliations:** ^1^Department of Neurology, Children’s Hospital Affiliated to Zhengzhou University, Henan Children’s Hospital, Zhengzhou Children’s Hospital, Zhengzhou, China; ^2^Beijing Chigene Translational Medicine Research Center Co., Ltd., Beijing, China; ^3^The Beijing Children’s Hospital Affiliated to Capital Medical University, Beijing, China

**Keywords:** SMC3 gene, MECP2 gene, PMP22 gene, clinical characteristics, dual-positive gene variations

## Abstract

**Objective:**

To analyze the clinical and genetic characteristics associated with dual-positive gene variations.

**Methods:**

A retrospective analysis was conducted on two children diagnosed with dual-positive gene variations.

**Results:**

Patient 1, a 7-year-old girl, presented with a low hairline, microcephaly, high-arched eyebrows, thick eyebrows, a short nasal bridge, a thin and red upper lip, and a high palatal arch. She exhibited delayed language and motor development. Genetic analysis revealed a *de novo* variation in the SMC3 and MECP2 genes. Patient 2, a 1.5-year-old boy, exhibited high-arched eyebrows, long eyelashes, large ears, microcephaly, a single transverse palmar crease, a curved fifth finger, tremors in the hands and feet, external rotation of both feet, and a staggering gait. He was unable to squat, had reduced muscle strength in the distal lower limbs, and electromyography suggested neurogenic damage. Additional findings included patent ductus arteriosus and mild auditory abnormalities. Genetic analysis identified a *de novo* variation in the SMC3 gene and a 1.38 Mb pathogenic haploid duplication at the PMP22 gene in both the proband and his father. The PMP22 gene duplication was also present in his cousin, aunt, and grandfather.

**Conclusion:**

We identified a rare case of a child with Cornelia de Lange Syndrome type 3 (CDLS3), accompanied by severe cognitive impairment, attributed to variations in the SMC3 and MECP2 genes. The MECP2 gene variation, while not resulting in Rett syndrome, may exacerbate the cognitive impairment. Additionally, we observed a rare instance of CDLS3 co-occurring with Charcot–Marie–Tooth disease type 1A. In situations where a single gene cannot be accounted for the clinical phenotype, it is imperative to consider the potential involvement of additional genetic variations.

## Introduction

Cornelia de Lange Syndrome (CDLS) is a rare congenital developmental disorder characterized by craniofacial malformations, growth and developmental abnormalities, limb malformations, cognitive impairment, and gastroesophageal reflux, among other symptoms (Li et al., 2021). Genetic variations associated with CDLS have been identified in several genes, including SMC1A, SMC3, RAD21, NIPBL, HDAC8, BRD4, and ANKRD11 (Cochran et al., 2019), with mutations in the NIPBL gene being the most prevalent (Kline et al., 2018). Instances of Cornelia de Lange type 3 (CDLS3) attributed to variations in the SMC3 gene are extremely rare. This study presents two cases of rare genetic variations: one involving SMC3 and MECP2 gene mutations, and the other involving SMC3 and PMP22 gene mutations, to enhance the understanding of these related rare diseases.

## Materials and methods

### Clinical data

The family members of the participants in this study signed the informed consent form.

### Genetic analyses

Sample Collection: Peripheral blood samples treated with EDTA were collected from patients after obtaining informed consent. DNA Extraction and Sequencing: Whole-exome sequencing was conducted using the BGI DNBSEQ-T7 platform (PE150). This sequencing was carried out by the Beijing Chigene Translational Medicine Research Center Co., Ltd., Beijing, China, with the postal code 100875. Subsequent data analysis was performed utilizing an online Genetic Diagnostic Platform provided by Chigene. The variants identified were classified in accordance with the Standards and Guidelines for the Interpretation of Sequence Variants issued by the American College of Medical Genetics and Genomics (ACMG) and the Association for Molecular Pathology.

### Clinical results

Patient 1: The subject was a 7-year-old female, gravida 2 para 2, delivered at full term via spontaneous delivery. Prenatal examinations revealed no abnormalities, and there was no history of perinatal hypoxia or asphyxia. Her birth weight was 3.4 kg, and her head circumference at birth was within normal limits. The clinical presentation included a low hairline, microcephaly with a head circumference of 49 cm, high-arched and thick eyebrows, a short nasal bridge, a thin and red upper lip, and a high palatal arch. The patient exhibited delayed language development, characterized by unclear speech; she was able to communicate with her parents by age 4 and could form short sentences of 6–7 words by age 7. Additional symptoms included drooling, hyperactivity, poor concentration, the ability to understand and follow simple instructions, the ability to eat with a spoon, and the ability to identify some body parts, with no evidence of language regression. Motor development was also delayed: the patient could hold her head up at 6 months, sit independently by 1 year, and walk by 2 years. She is currently able to run but cannot jump high with both feet or stand on one foot. Feeding difficulties were observed during infancy. There was no evidence of stereotypical hand movements or limb deformities, and muscle strength and tone in the limbs were normal. Importantly, there was no regression in motor development. Additional assessments, including complete blood count, urinalysis, and biochemical tests, revealed normal results. Evaluations of inorganic elements and thyroid function, as well as metabolic screenings of blood and urine, were also within normal limits. Both the standard electroencephalogram and cranial MRI showed no abnormalities. However, her gross motor and fine motor skills demonstrate mild developmental delays, but her adaptive, language, and personal–social skills all demonstrate moderate developmental delays. The Gesell Developmental Scale assessment conducted at age 3 indicated developmental functioning equivalent to that of a 1-year and 7-month-old child. Furthermore, the Autism Behavior Checklist (ABC)and the Social Responsiveness Scale (SRS) assessments suggested a borderline autism spectrum condition.

**Figure 1:**
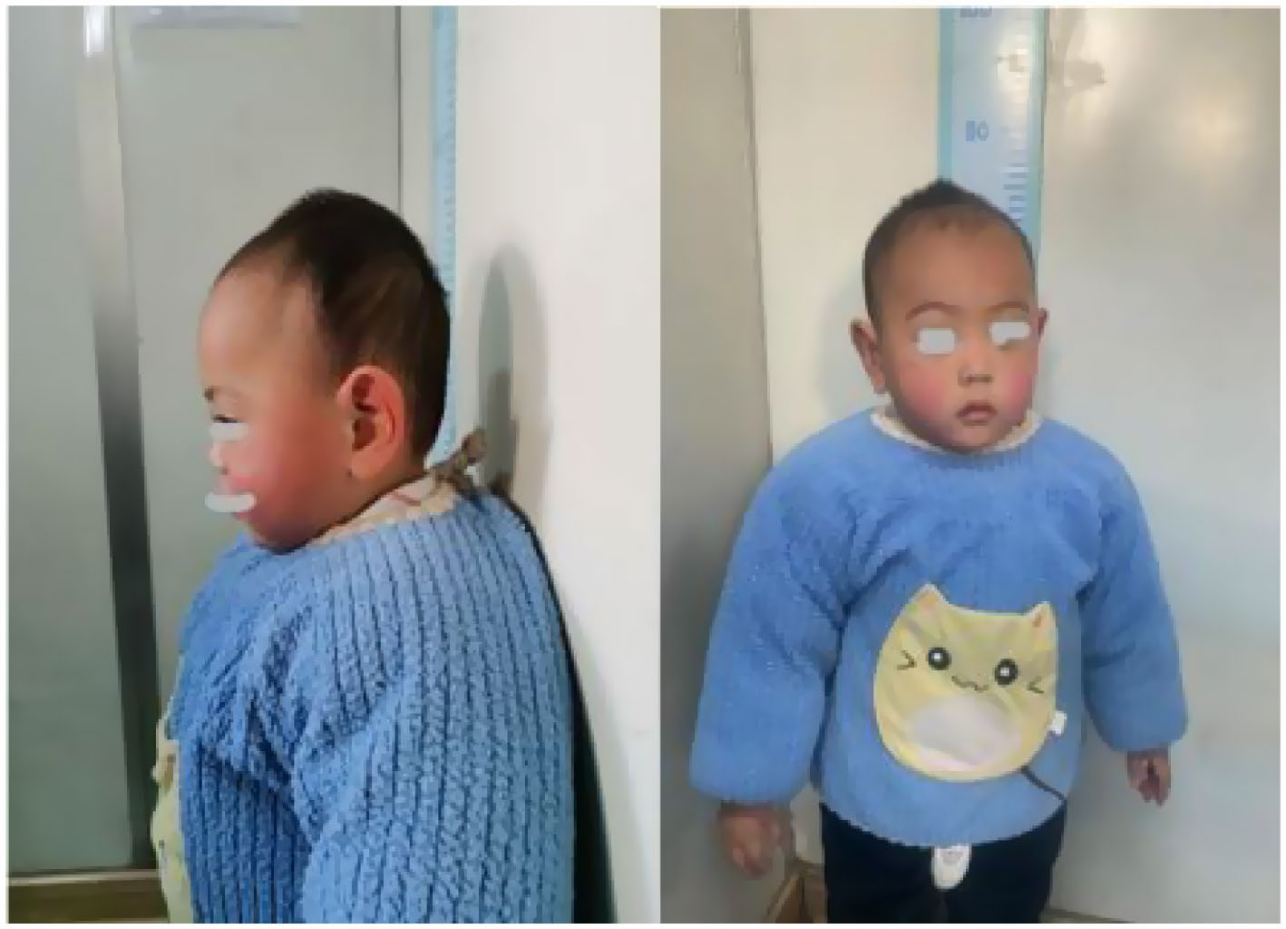
Craniofacial features of patient 2: high-arched eyebrows, long eyelashes, microcephaly.

**Figure 2:**
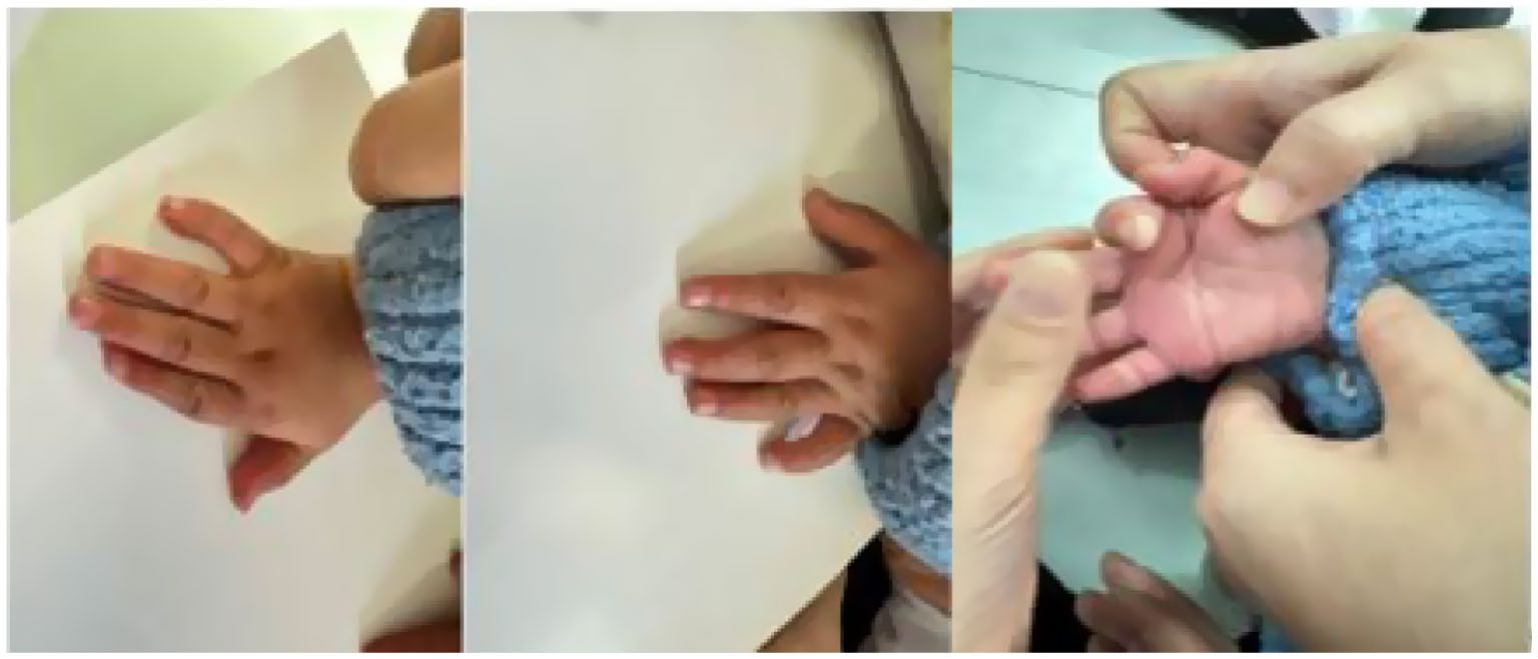
The fifth little finger of the left and right hands of patient 2 has bilateral fifth-digit clinodactyly and simian creases.

Patient 2: The subject was a male child aged 1 year and 6 months, with a birth history of G1P1, delivered via cesarean section due to premature rupture of membranes at 28 weeks of gestation. There was no history of perinatal hypoxia, and the child’s head circumference, height, and weight at birth were consistent with developmental expectations for 28 weeks of gestation. Clinically, the patient presented with distinctive features, including high-arched eyebrows, long eyelashes, large ears, and microcephaly (refer to Figure 1). The head circumference measured 45 cm, and the height was 81 cm. Additional physical observations included a single transverse palmar crease, clinodactyly of the fifth digit (refer to Figure 2), hand and foot tremors, external rotation of both feet, and a staggering gait. The patient was unable to squat, although limb muscle tone appeared normal, and distal limb muscle strength was assessed as 5-. Language development was delayed, as evidenced by his inability to communicate with his parents. Auxiliary diagnostic evaluations revealed normal results for routine hematological and biochemical tests, as well as normal findings in blood and urine metabolic screenings. The intelligence assessment indicated cognitive functioning equivalent to that of an 11-month-old child. Electromyographic analysis suggested neurogenic damage, with an upper limb motor nerve conduction velocity (NCV) of less than 38 m/s. Color Doppler echocardiography identified a patent ductus arteriosus. The MRI of the head revealed a cavum septum pellucidum, as well as a septum pellucidum cyst. The cystic structure adjacent to the septum pellucidum was identified as a non-specific imaging alteration.

### Genetic results

**Figure 3:**
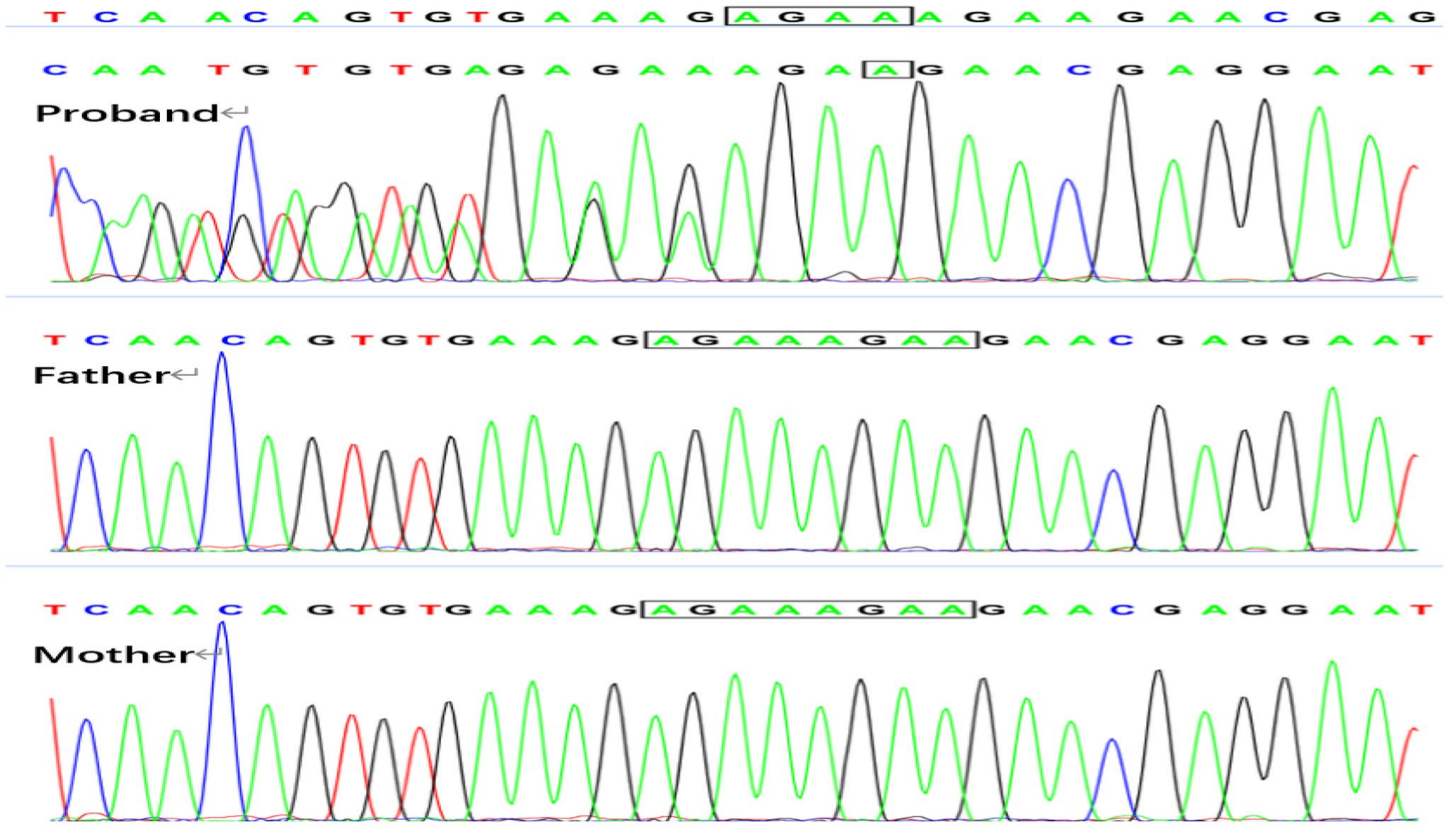
SMC3 gene: c.1071 1074delAGAA, both the father and mother of the proband are wild type.

**Figure 4:**
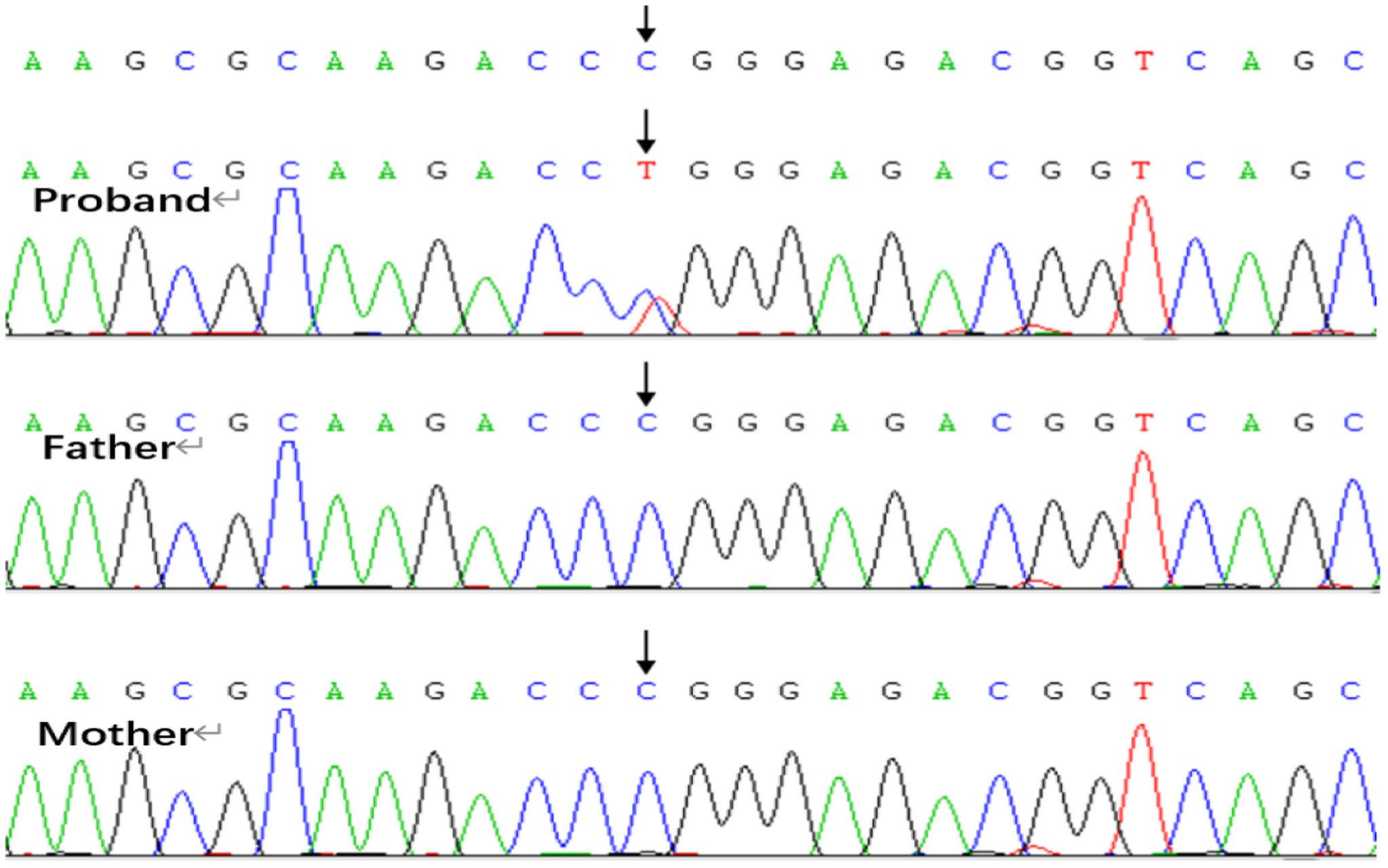
MECP2 gene: c.961C > T in patient 1, and both the father and mother proband are wild type.

Genetic analysis revealed *de novo* variations in the SMC3 and MECP2 genes in Patient 1. The identified variation in the SMC3 gene (Figure 3) is c.1071_1074delAGAA, p.E358Nfs*44 (p.Glu358Asnfs*44). This alteration involves the deletion of the nucleotide sequence AGAA between positions 1,071 and 1,074 in the SMC3 gene’s coding region, leading to a frameshift mutation. As a result, the protein sequence is altered from the 358th amino acid, with glutamic acid (Glu) being replaced by asparagine (Asn), and the protein sequence terminating after an extension to the 44th position. This variant is cataloged in the ClinVar database as pathogenic (Variation ID: 626275) and is classified as pathogenic according to ACMG guidelines, with criteria PVS1, PS2, and PM2. Variations in the SMC3 gene are associated with Cornelia de Lange syndrome type 3, which aligns with the clinical symptoms observed in the patient. The *de novo* variation in the MECP2 gene, illustrated in Figure 4 as c.961C > T, p.R321W (p.Arg321Trp), was identified, with both parents exhibiting the wild-type genotype. This variation results in the substitution of a cytosine nucleotide (C) with a thymine nucleotide (T) at position 961 of the MECP2 gene’s coding sequence. Consequently, the amino acid at position 321 of the protein is altered from arginine (Arg)to tryptophan (Trp), constituting a missense variation. This specific variation is cataloged in the ClinVar database as a pathogenic variant. Furthermore, according to the American College of Medical Genetics and Genomics (ACMG) guidelines, it is classified as likely pathogenic, with the following criteria: PS2, PM2_Supporting, and PP3.

**Figure 5:**
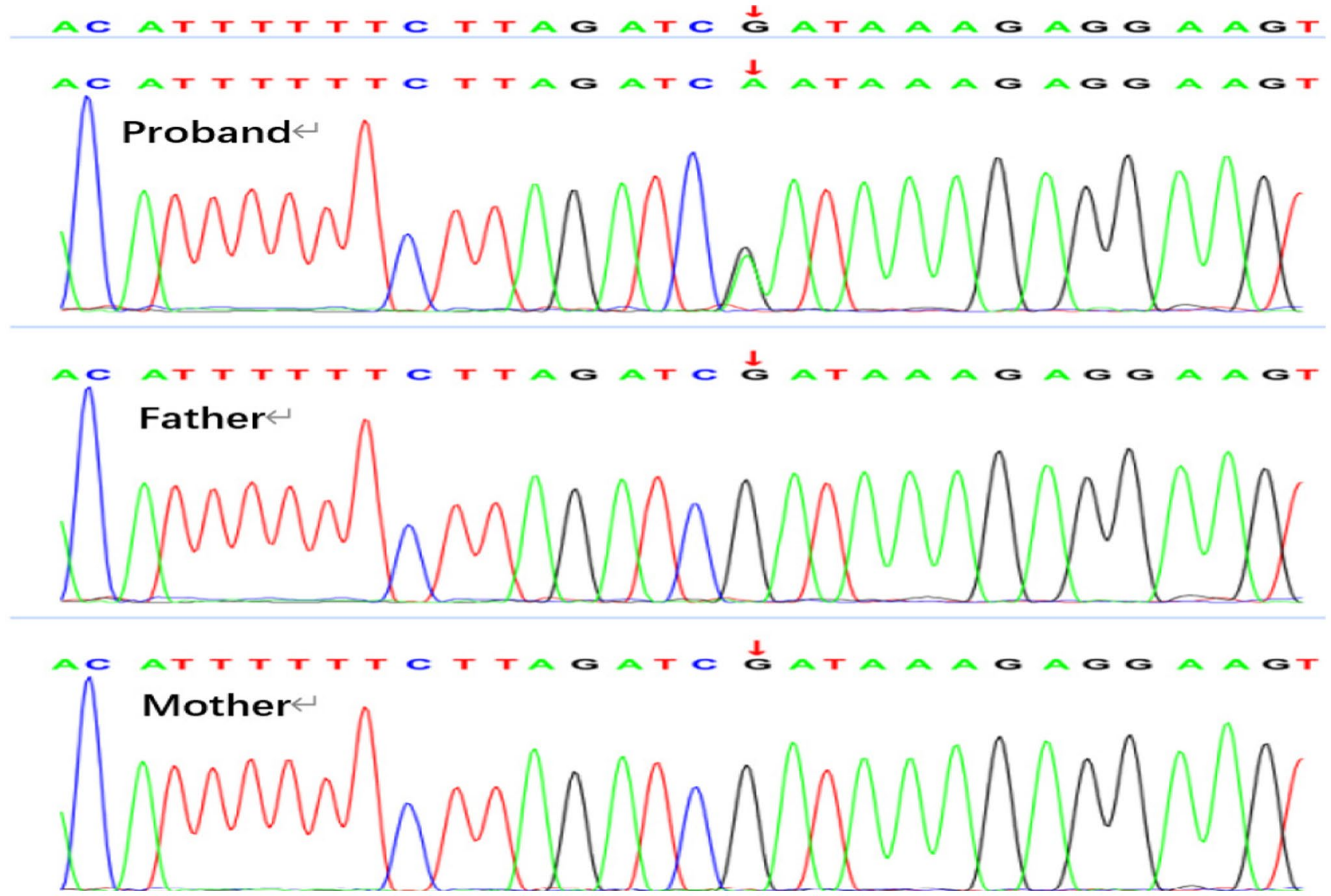
SMC3 gene: c.274 (exon6) G > A, both father and mother of the proband are wild type.

**Figure 6:**
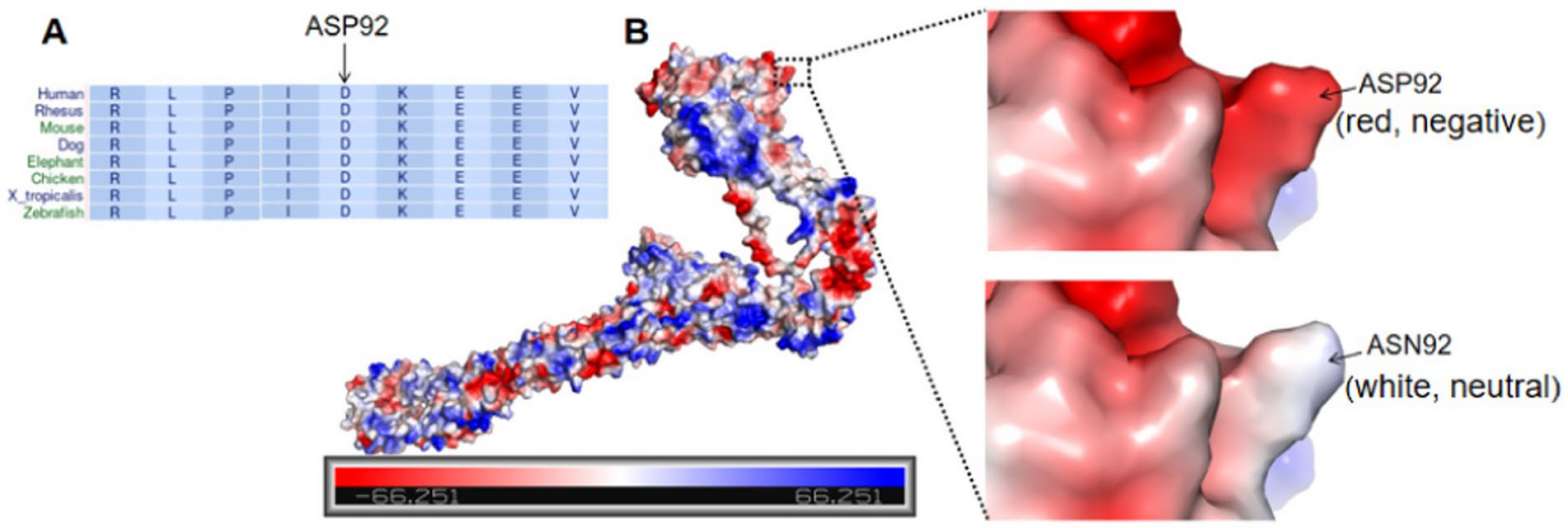
**(A)**.Conservation analysis among nine species showed p.Asp92Asn variation happened in a highly conserved region of SMC3 among different species. **(B)**.Variation of negatively charged Asp-92 to a neutral Asn residue causes changes in electrostatic charge distribution, which may affect the protein function. Proteins are shown with electrostatic surface (The colors red, white and blue indicate negative, neutral and positive charges, respectively).

**Figure 7:**
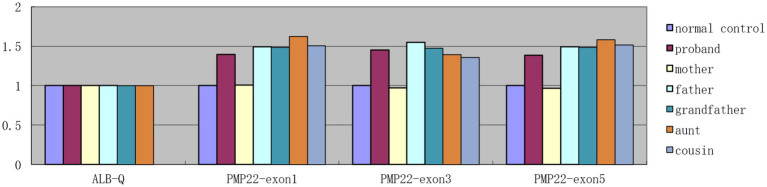
PMP22: gain1 (EXON: 1-5) in the patient 2 and father, mother, cousin, aunt, and grandfather of patient 2.

Genetic analysis revealed a *de novo* variant in the SMC3 gene in Patient 2, specifically c.274G > A, p.D92N (p.Asp92Asn), while both parents exhibited the wild-type allele (Figure 5). According to the American College of Medical Genetics and Genomics (ACMG) guidelines, this variant is classified as likely pathogenic, with evidence codes PS2, PM1, PM2_Supporting, PP2, and PP3. Conservation analysis across nine species indicated that the p.Asp92Asn variant occurs in a highly conserved region of SMC3, suggesting its potential impact on protein function. The substitution of the negatively charged aspartic acid (Asp-92) with a neutral asparagine (Asn) residue alters the electrostatic charge distribution, which may affect protein function (Figure 6). Patients harboring pathogenic variants in SMC3 are known to develop Cornelia de Lange syndrome type 3, which aligns with the clinical symptoms observed in Patient 2. Additionally, copy number variation (CNV) analysis revealed an approximately 1.38 Mb pathogenic haploid duplication at the chr17:14095305–15472344 locus in both the proband and his father, while the mother remained wild-type. This duplicated segment includes the PMP22 gene. The cousin, aunt, and grandfather of the affected individuals within the tested family exhibited high foot arches and abnormal gait patterns in the lower limbs. All were found to carry the PMP22 gene duplication, as confirmed by quantitative polymerase chain reaction (q-PCR) (Figure 7). The duplication of the PMP22 gene is known to cause Charcot–Marie–Tooth disease (CMT).

## Discussion

The incidence of Cornelia de Lange Syndrome (CdLS) in neonates is approximately 1 in 10,000 to 30,000 (Bottai et al., 2019), with inheritance patterns predominantly autosomal dominant or X-linked. The clinical spectrum of CdLS varies from mild to severe manifestations. The classical presentation of CdLS is characterized by distinctive facial features, growth retardation, hirsutism, and anomalies of the upper limbs and hands. Individuals exhibiting milder phenotypes demonstrate less pronounced growth, cognitive, and limb involvement. Additional common clinical manifestations include cardiac defects, gastrointestinal dysfunction, hearing impairment, myopia, cryptorchidism, and genital hypoplasia, among others (Deschamps, 2022). Advances in genetic technology have facilitated the identification of novel genetic variants associated with CdLS, beyond the previously recognized genes such as SMC1A, SMC3, RAD21, NIPBL, HDAC8, BRD4, and ANKRD11. Newly identified genes include MAU2 (Ascaso et al., 2024), KMT2D, KDM6A, and UBE2A, among others (Shangguan and Chen, 2022). A comprehensive literature review utilizing databases such as Wanfang, CNKI, VIP, the Chinese Medical Association's databases, PubMed, and Web of Science revealed a total of 62 reported cases of CdLS attributable to SMC3 mutations globally, with 27 cases providing detailed clinical histories. *de novo* pathogenic variants in SMC3 account for approximately 1–2% of Cornelia de Lange syndrome (CdLS) cases. The associated phenotype is characterized by postnatal-onset microcephaly, subtle craniofacial dysmorphism, and relatively mild prenatal growth retardation that progresses to more significant postnatal growth failure. Notably, these individuals exhibit infrequent occurrence of congenital structural cardiac anomalies and an absence of limb defects (Kaur et al., 2023). In this study, both children exhibited microcephaly, with no evidence of growth retardation detected during prenatal examinations, aligning with previous reports. The facial characteristics of the two children were relatively typical. Patient 1 presented with a low hairline, high-arched and thick eyebrows, a short nasal bridge, and a high palatal arch, whereas Patient 2 exhibited high-arched eyebrows, long eyelashes, prominent ears, and low ear positioning, which deviated from the aforementioned reports. Cardiac ultrasound results for Patient 1 were normal, while Patient 2 displayed a patent ductus arteriosus. Additionally, Patient 2 had a single transverse palmar crease and a clinodactyly of the fifth finger, whereas Patient 1 showed no limb development abnormalities. These observations underscore the fact that identical genetic variations can result in diverse clinical phenotypes, highlighting the heterogeneity of clinical presentations. A domestic report previously described a case of fetal hand deformity associated with the c.3298G>A missense variation in the SMC3 gene (Huang et al., 2024). In contrast, our two subjects exhibited no significant limb-related deformities during prenatal examinations.

In this study, Patient 1 exhibited dual-positive variations in the SMC3 and MECP2 genes. The MECP2 gene is predominantly associated with Rett syndrome, which is categorized into two forms: typical and atypical. Notably, this patient did not exhibit hallmark symptoms of Rett syndrome, such as stereotypical hand movements, loss of language function, abnormal gait, epileptic seizures, developmental regression, or respiratory and sleep abnormalities. According to the expert consensus on the international diagnostic criteria for Rett syndrome established in 2010, this patient did not fulfill the criteria for either typical or atypical Rett syndrome (Neul et al., 2010). Literature indicates that MECP2 gene variations have been identified in individuals without Rett syndrome, including a female asymptomatic carrier within a Rett syndrome family, a phenomenon attributed to extreme skewing of X chromosome inactivation (Wan et al., 1999). Individuals with MECP2 variations may present with other neurodevelopmental disorders, including autism, Angelman syndrome-like manifestations, and nonspecific intellectual disabilities (Carney et al., 2003; Watson et al., 2001). The splicing variant c.2535 + 1G > A in the SMC3 gene results in a spectrum of severe clinical phenotypes (Lei et al., 2024). In contrast, missense variants in the NIPBL and SMC3 genes are typically associated with milder clinical manifestations (Zhu and Wang, 2018). Among the six reported cases of amino acid frameshift variations caused by nucleotide deletions in the SMC3 gene with detailed clinical data, five exhibited mild to moderate clinical phenotypes. Most variations in the SMC3 gene are associated with mild to moderate cognitive developmental impairments (Li et al., 2021). However, the subject of this study presents with severe intellectual disability. Currently 14 years old, the subject exhibits language developmental disorders, can articulate only short sentences of six to seven words, experiences drooling, displays hyperactivity, has poor concentration, and can comprehend and follow only simple instructions. It is hypothesized that these severe manifestations may be attributed to the combined effects of variations in the SMC3 and MECP2 genes. Charcot–Marie–Tooth disease (CMT) represents the most prevalent monogenic hereditary disorder affecting the peripheral nerves, with an estimated prevalence of approximately 40 per 100,000 individuals. The onset of CMT typically occurs during childhood or adolescence, and its clinical manifestations include chronic progressive symmetrical muscle weakness and atrophy, distal sensory impairment, diminished or absent tendon reflexes, pes cavus, and other skeletal deformities (Pareyson and Marchesi, 2009). The demyelinating form of CMT is characterized by a marked reduction in nerve conduction velocity (NCV), with the motor NCV in the upper limbs generally measuring less than 38 m/s. Pathological examination of nerve biopsies often reveals significant abnormalities in peripheral nerve myelin (Yao and He, 2024). CMT1, an autosomal dominant demyelinating form of CMT, is the most prevalent type. Within this category, CMT1A is the most common subtype, accounting for 40 to 50% of all CMT cases. The majority of CMT1A cases are associated with a duplication of a 1.4 Mb segment on chromosome 17p11.2-12, which includes the peripheral myelin protein 22 (PMP22) gene (Lupski et al., 1992). The CNV analysis for Patient 2 revealed a pathogenic haploid duplication of approximately 1.38 Mb at the chromosomal location chr17:14095305–15472344. The mother was identified as wild-type, while the duplicated segment included the PMP22 gene. Verification through quantitative polymerase chain reaction (q-PCR) demonstrated that the cousin, aunt, father, and grandfather of the affected family members also possessed the PMP22 gene duplication. These individuals exhibited phenotypic characteristics such as high foot arches and abnormal gait. Notably, the grandfather, father, and aunt displayed the ability to stand on one leg, reminiscent of a crane posture, in the lower limbs, consistent with familial genetic co-segregation. Electromyography of the proband indicated a nerve conduction velocity (NCV) of the limbs at less than 38 m/s. Considering the clinical manifestations in the child, including hand and foot tremors, external foot rotation, a staggering gait, inability to squat, and distal limb muscle strength rated at 5-, alongside the electromyography and genetic findings, a diagnosis of Charcot–Marie–Tooth disease (CMT) was established, specifically aligning with the CMT1A subtype. Additionally, Patient 2 exhibited a *de novo* mutation in the SMC3 gene and was diagnosed with Cornelia de Lange Syndrome type 3 (CdLS3) based on the clinical phenotype. This child presented with two rare genetic conditions. Iran reports a case with concomitant variants: a novel homozygous HERC1 gene variant and a novel heterozygous PMP22 duplication (Reshadmanesh et al., 2024). CAPRA (a probabilistic risk assessment platform) and adverse pathology (AP) report three unrelated cases of dual diagnosis (Capra et al., 2023). Consequently, when the clinical phenotype of a child cannot be attributed to a single gene, it becomes imperative to consider the possibility of additional genetic abnormalities.

## Conclusion

In conclusion, our study identified two pediatric cases exhibiting rare diseases attributable to dual-positive genetic variations. In the first case, the patient presented with Cornelia de Lange syndrome type 3, accompanied by severe cognitive impairment, resulting from variations in the SMC3 and MECP2 genes. It is posited that the non-Rett syndrome phenotype, associated with the MECP2 gene variation, may exacerbate the cognitive impairment observed in this patient. The second case involved a patient with Cornelia de Lange syndrome type 3, co-occurring with Charcot–Marie–Tooth disease type 1A, due to an SMC3 gene variation and PMP22 gene duplication. These findings underscore the necessity of considering additional genetic variations when a single gene cannot be fully explain a clinical phenotype. This approach can enhance the foundation for genetic counseling for affected families and inform strategies for eugenics, as well as optimal prenatal and postnatal care. Due to the small number of cases, this article has certain limitations and requires more case studies to fully describe the clinical characteristics of this gene mutation.

## Data Availability

The datasets presented in this study can be found in online repositories. The names of the repository/repositories and accession number(s) can be found in the article/supplementary material.
